# Scouting the receptor-binding domain of SARS coronavirus 2: a comprehensive immunoinformatics inquisition

**DOI:** 10.2217/fvl-2020-0269

**Published:** 2021-02-22

**Authors:** Zaira Rehman, Ammad Fahim, Muhammad Faraz Bhatti

**Affiliations:** 1 ^1^Department of Virology, National Institute of Health (NIH), Islamabad, Pakistan; 2 ^2^Department of Multidisciplinary Sciences, National University of Medical Sciences (NUMS), Rawalpindi, Pakistan

**Keywords:** coronaviruses, COVID-19, epitope mapping, human ACE2 receptor

## Abstract

**Aim:** December 2019 witnessed the emergence of a worldwide outbreak of a novel strain of coronavirus (CoV) termed SARS-CoV-2. Several preventive strategies are being developed, such as vaccines, to stop the spread of infection. **Materials & methods:** A comprehensive immunoinformatics approach was used to map conserved peptide sequences on the receptor binding domain of SARS-CoV-2 for their B-cell, T-helper & T-cytotoxic cell epitope profiles. **Results & conclusion:** The antigenic B-cell epitopes were LFRKSN and SYGFQPT. Among T-cell epitopes, CVADYSVLY and FTNVYADSF exhibited affinity for MHC class I, while YRLFRKSNL and VYAWNRKRI exhibited affinity for of MHC class II alleles. The overlapping epitope between B- and T-cells was YRLFRKSNL. The deployment of these epitopes in potential vaccine development against COVID-19 may help in slowing down the SARS-CoV-2 spread.

The COVID-19 pandemic [1] with its widespread pathogenesis on human body reminds us of the SARS and Middle East respiratory syndrome (MERS) epidemics [2,3]. Before the emergence of SARS family-mediated epidemics, coronaviruses (CoVs) were not a notable pathogenic entity, although they have been cited in the literature since the 1960s [4]. Besides SARS-CoV-2, the SARS-CoV and MERS-CoV were other famous coronaviral strains known for crossing the species barrier and infecting humans, while 229E, NL63(alpha), OC43 and HKU1 (beta) strains of CoVs caused common cold in humans [5]. The SARS-CoV that surfaced during 2002–2003 in China, infected almost 8000 individuals and caused 774 causalities in 37 countries [6,7] while MERS-CoV emerged in 2012 and infected 2494 humans causing 858 mortalities [2,8,9]. The ongoing epidemic of SARS-CoV-2 has affected more than 13 million and caused 0.5 million deaths globally up to 15 July 2020 [10]. The detailed viral sequence of SARS-CoV-2 indicates a genetic similarity to its earlier family members SARS and MERS, harboring six open reading frames [11]. The genome of CoVs is generally 27–35 kb, stuffed inside a nucleocapsid protein envelope. There are three structural proteins associated with viral envelope: membrane, envelope and spike (S) proteins [12]. The S protein is essential for viral entry into host but also impels tissue tropism, host diversity as well as host immune responses [13]. The S protein constitutes S1 and S2 subunits where S1 harbors receptor-binding domain (RBD) while S2 aids in virus–host cell fusion. Interestingly, the S gene of CoVs has been noticeable for the most recurring recombination breakpoints in SARS-CoV [14]. In case of SARS CoV, the main entry receptor on host cell is ACE2 [15] while in case of MERS-CoV, the primary entry receptor is dipeptidyl peptidase 4 (also known as CD26) [16]. Comparatively, the genomes of SARS-CoV and MERS-CoV although harboring significant similarity, they potentially harbor variation, particularly from the antigenic response they might generate with reference to RBD [17].

Receptor-binding protein has been previously targeted for vaccinable solutions [18,19]. However, from the evolutionary standpoint, coronaviral RBD is reported as a hypervariable region [20]. There are no approved treatments or vaccines available to tackle COVID-19 thus far; however, development has been fast tracked. Contextually, vaccinomics approach exploits immunogenomics of SARS-CoV-2 RBD that can lead to potential vaccine candidate identification. Immunoinformatics driven in-depth analysis can aid in identifying repertoire of viral antigenic epitopes that may be either linear or discontinuous and it also helps in identifying whether these epitopes are immunogenic or virulent [21]. A comprehensive immunoinformatics data mining of SARS-CoV-2 RBD can increase our understanding for its antigenic profile. Encouragingly enough, epitope prediction analyses were reported earlier for SARS and MERS strains [19,22] and more recently for SARS-CoV-2 [23–25].

Numerous studies have reported bats as the primary reservoirs of SARS and MERS viruses [14,26–29]; however, rodent origin has also been reported [13,30]. A recently reported strain derived from bats, the bat CoV (BatCoV RaTG13) shares more than 96% homology with SARS-CoV-2 and 93% with its S protein, rendering it to be a close relative of SARS-CoV-2 [11]. Anatomically, conservative peptide sequence of coronaviral RBD compared with the closest known zoonotic coronaviral strain can provide better potential vaccine candidates for human testing. After the emergence of COVID-19 pandemic, SARS-CoV-2 surface protein has been repeatedly utilized for the identification of potential vaccine epitopes for SARS-CoV-2 [23–25]. However, there has also been simultaneous speculation regarding potential existence of cross resistance epitopes between SARS-CoV-2 and SARS-CoV [17,31,32]. Earlier, the computational probing of protein structures for respiratory infections via employment of docking methods has added useful information to stereochemical properties, virus binding mediated host receptor conformational transformation and binding preferences [33–37]. Contextually, viral receptor interactions were considered valuable in the instances of picornaviruses, influenza, HIV and CoVs [20,38–40]. For the SARS-CoV-2-induced infection, the basic reproduction number for viral transmissibility (R_o_) as per various estimates is around 1.1–5.5 [41,42]. Since this threshold suggests very high infectivity rate, it is pertinent to target the viral binding region with vaccines to prevent infection.

Vaccinable peptide sequence for epitope based vaccine in case of alphaviruses, hepatitis B and C, HIV, HPV, and influenza viruses for recognition of potent immunogens has given propitious results [43–46]. Several studies reported on SARS and MERS CoV strains provided useful information regarding the potential epitopes retained by these strains [47–54], while, the data in context pertinent to SARS-CoV-2 are insufficient. The global COVID-19 pandemic has sparked rigorous R&D activity for vaccine development, and in a matter of just 4 months, various potential vaccine candidates are in the preclinical and clinical development phases [55]. The clinical behavior of SARS-CoV-2, infecting people around the world, with varied clinical symptomatology, ranging from completely asymptomatic to rapidly progressing lethal respiratory insufficiency demands the utilization of thorough and rapid novel technology platforms with more vaccinable options against SARS-CoV-2 [55,56].

This study reports key findings vis-a-vis SARS-CoV-2 RBD for its variable and conservative residues in comparison with BatCoV RaTG13 strain, which can be considered immunogenic epitopes for potential multi-epitope vaccine candidate for SARS-CoV-2 in the backdrop of its binding orientation.

## Materials & methods

**Figure 1.**
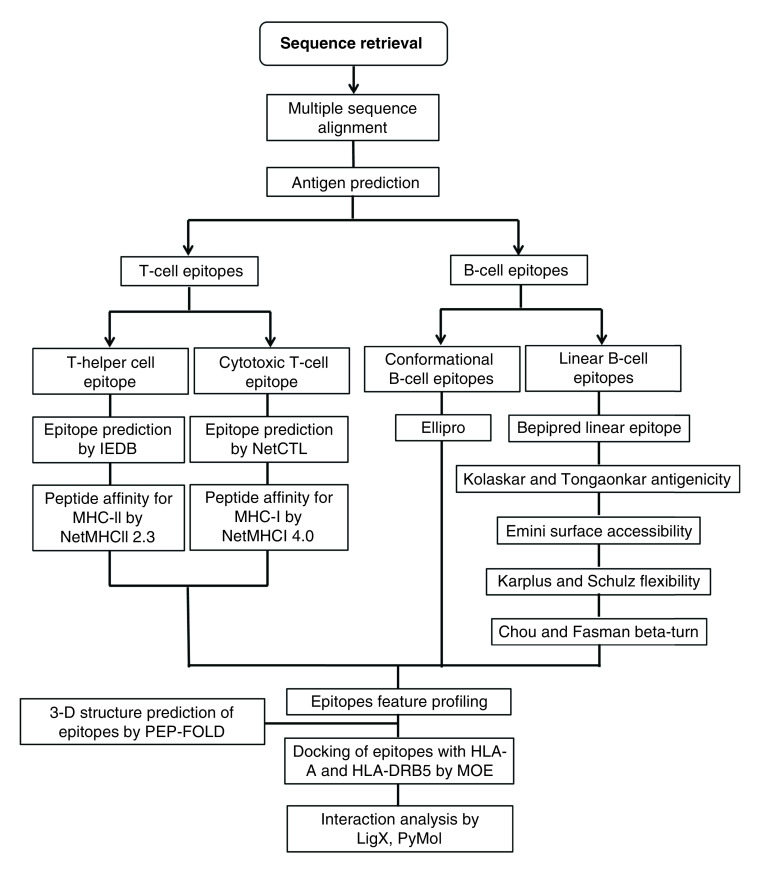
Detailed workflow for analysis of receptor binding domain of SARS coronavirus 2. The sequences of SARS-CoV-2, BatCoV RaTG13 and SARS-CoV were downloaded from the NCBI followed by multiple sequence alignment through ClustalX and JalView. Within the receptor-binding domain, the antigenic epitopes were also determined using immune-informatics approach. Linear and conformational B-cell epitopes were determined. Linear B-cell epitopes were determined through different methods that included BepiPred, Karplus & Schulz, Kolaskar & Tongaonkar, Emini, and Chou & Fasman methods. Conformational B-cell epitopes were determined through Ellipro. Cytotoxic and helper T-cell epitopes were also determined. CTL epitopes were determined through NetCTL followed by their affinity for specific MHC-I alleles through NetMHCI 4.0. Helper T-cell epitopes were identified through IEDB followed by identification of allele specificity through NetMHCII 2.3. All the B- and T-cell epitopes were then subjected to feature profiling by determining their toxicity, mutation, antigenicity, conservation, charge, molecular weight and nondigesting enzymes. Selected T-cell epitopes were then modeled through PEPFOLD. The interaction analysis of peptides with HLA-A and HLA-DR was done through MOE. Interacting residues of HLA-A and HLA-DR with peptides were determined through LigX in MOE and PyMOL. BatCoV: Bat CoV; CoV: Coronavirus; CTL: Cytotoxic T lymphocyte; IEDB: Immune epitope database; MHC-I: MHC class I; MOE: Molecular operating environment; NCBI: National Center for Biotechnology Information.

To identify the presence of antigenic epitopes within the RBD of S glycoprotein of SARS-CoV-2, *in silico* analysis was performed (Figure 1). The antigenicity of RBD was determined through VaxiJen v2.0. It is an alignment-free method for prediction of antigenicity. It predicts the antigenicity of proteins on the basis of physicochemical properties of amino acids [57].

## Sequence retrieval & multiple sequence alignment

Protein sequences of S glycoprotein of SARS-CoV-2 (reported till 31 March 2020) were retrieved from National Center for Biotechnology Information. The sequences reported from China, Australia, USA, Taiwan, India, Pakistan, Nepal, Italy, Sweden, Brazil, Vietnam, Spain, Colombia, Peru and Japan were selected for analysis. The sequence of S glycoprotein from BatCoV RaTG13 (GISAID accession no. EPI_ISL_402131) and SARS CoV ZJ02 (Accession No. ABB29898) were used as reference for comparison. Sequence analysis was performed to ascertain the changes in the RBD of S glycoprotein. Multiple sequence alignments were performed using Clustal X. The consensus sequence of SARS-CoV-2 was used as input for epitope prediction.

### B-cell epitope prediction

For identification of B-cell epitopes, Immune Epitope Database (IEDB) and BepiPred-2.0 were used [58,59]. The consensus sequence of SARS-CoV-2 was used to predict B-cell epitopes through Karplus & Schulz flexibility, Kolaskar & Tongaonkar antigenicity, Chou & Fasman beta-turn, Emini surface accessibility and BepiPred linear epitope prediction methods. Kolaskar & Tongaonkar method predicted antigenicity on the basis of amino acid abundances in naturally occurring epitopes as well as their physicochemical properties. The default threshold was set to 1.00 for antigenicity determination [60]. Emini surface accessibility method predicts the surface accessibility of epitopes as the surface accessible peptides recognized by the immune system [61]. Chou & Fasman beta-turn method was used to predict the antigenic regions, exhibiting beta turn as the beta turns are usually hydrophilic in nature and highly accessible [62]. Karplus & Schulz flexibility method predicts those antigens that are exhibiting flexible amino acids in nature as flexibility is correlated with antigenicity [63]. BepiPred prediction method based on hidden Markov model predicts linear epitopes in protein [64]. The B-cell epitopes were also predicted using ElliPro, which identifies linear and discontinuous epitopes in protein structure. It calculates the protrusion index (PI) of the residues and then cluster the residues on the basis of protrusion index values [65].

### T-cell epitope prediction

For vaccine development, cytotoxic T-lymphocyte (CTL) epitopes play an important role. Hence the T-cell epitopes were identified that have the ability to bind with MHC class I (MHC-I) and class II (MHC-II). CTL epitopes were identified through NetCTL 1.2 server. It is based on artificial neural network and trained on different human MHC alleles for prediction of epitopes [66]. The IEDB and NetMHCI 4.0 server were used to predict the binding of epitopes with MHC-I. NetMHCI 4.0 predicts the binding affinity through artificial neural network by schooling 81 distinct HLA-A, -B, -C and -E human MHC alleles [67]. T-helper cell epitopes were predicted through IEDB and NetMHCII 2.3 server [68]. The epitopes were predicted having high affinity toward HLA-DR, -DQ and -DP. The length of the predicted epitopes was set to be 9-mer epitopes as it is reported that most HLA molecules have a strong preference for binding 9-mer epitopes [33]. For all the T-cell epitopes the threshold for predicting the binding affinity was set to ≤500 nM.

### B- & T-cell epitopes feature profiling

B- and T-cell epitopes were further scrutinized for their enzymatic digestion, toxicity, hydrophobicity and physiochemical properties. The digestion of peptides with enzymes is an important parameter in vaccine development as the peptides that are digested by many enzymes are usually rendered unstable. Hence, the digestion of peptides by different enzymes was predicted through protein-digest server. AntiangioPred was used to predict the mutational variability and other physicochemical properties of peptides. It is based on machine learning model that is generated on the basis of already reported anti-angiogenic peptide [69]. ClanTOX predicted toxicity of peptides [70]. It is trained on set of ion-channel inhibitors and a set of nontoxin proteins. Based on the model, the program predicts the toxicity of input peptide. Antigenicity of peptides was predicted through Immunomedicine group server. For a peptide to be antigenic the threshold is 1.0. Antigenic prediction is based on physicochemical properties of epitopes like hydrophilicity, flexibility and accessibility [60].

### Human proteome analysis for nonhuman homologues

To avert autoimmunity, vaccine contenders were screened for human and nonhuman homologues. The nonhuman homologues were identified by scrutinizing selected epitopes sharing <30% identity with human proteome, via BLASTp analysis.

### Docking of T-cell epitopes with MHC-I & MHC-II alleles

The peptides that showed affinity for maximum number of MHC-I and MHC-II alleles were selected for interaction analysis. The structure of peptides was modeled through PEPFOLD server [71] followed by energy minimization. In case of MHC-I, the common allele between the peptides was selected for docking. Hence, the crystal structure of human HLA-A*0101 was downloaded from Protein Databank (PDB; PDB ID: 6AT9: resolution = 2.9 Å). Same criterion was followed for MHC-II alleles and for that the crystal structure of HLA-DRB5*01:01 (PDB ID: 1FV1: resolution = 1.9 Å) was also downloaded from PDB. Both the structures after ligand removal underwent protonation followed by energy minimization by AMBER99 force field. In order to analyze the inhibitory potential of peptides the docking of MHC-I specific peptides was executed with the HLA-A*0101 alleles, while the MHC-II specific peptides were docked with HLA-DRB5_0101. The docking studies were accomplished with induced fit docking protocol implemented in molecular operating environment (MOE) version 2016.08. By using, triangle match as placement method with London-dG scoring and GBVI/WSA dG rescoring function, 50 poses of each peptide were generated. Interaction analysis was done using MOE and PyMOL v2.3.

### Interaction analysis of BatCoV RaTG13 with bat ACE2

After identification of vaccine epitopes, we further explored whether these vaccine epitopes harbor any important residues that could be involved in binding of SARS-CoV-2 with human ACE2 (hACE2) and BatCoV RaTG13 with bat ACE2 (bACE2). The interactions of SARS-CoV-2 with hACE2 were recently reported by Lan *et al* [72]. The interaction analysis of BatCoV RaTG13 with bACE2 was performed in the current study. To the best of our knowledge, the structure of RBD of BatCoV RaTG13 as well as bACE2 has not been determined yet. Hence the structure of both the proteins was determined through homology modeling using Modeller V9.23. BatCoV RaTG13 was modeled using SARS-CoV as template (PDB ID: 2GHV). While bACE2 was modeled using hACE (PDB ID: 1R42) as template. The generated model was subjected to model evaluation and structural validation via Ramachandran plot, PROSA, ERRAT, QMEAN and MolProbity. Ramachandran plot calculates the presence of amino acid residues in allowed, favored and outlier regions on the basis of torsional angles (Ψ and Φ) of amino acids [73]. PROSA reveal the quality of model by estimating any error in the models. It also calculates the score of model on the basis of experimentally reported (x-ray and nuclear magnetic resonance) structures of proteins [74]. Qualitative model energy analysis (QMEAN) apprises the geometry of protein structure by measuring the torsion angles on three consecutive amino acid residues [75]. MolProbity evaluates the protein structure by assessing its geometry [76]. ERRAT gauged the quality of model by analyzing the statistics between nonbonded interactions and different type of atoms and compared these values with the extremely refined structures [77]. The best model was then subjected to energy minimization using AMBER99 force field implemented in MOE. Docking of BatCoV RaTG13:bACE2 was performed using HADDOCK web server [78]. Analysis of protein–protein interactions was performed through pdbSum [79] and PyMOL v2.3.

## Results

### Multiple sequence alignment

RBD of SARS-CoV-2 is 192 amino acids long (within position 330–522 amino acids) lying in S1 region of S glycoprotein. When comparing the receptor binding motif with the BatCoV RaTG13 there was variation between the two virus strains (Supplementary Figure 1). The important changes were observed at position 439 (Lys→Asn), 440 (His→Asn), 441 (Ile→Leu), 443 (Thr→Lys), 445 (Glu→Val), 449 (Phe→Tyr), 459 (Ala→Ser), 478 (Lys→Thr), 483 (Gln→Val), 484 (Thr→Glu), 486 (Leu→Phe), 490 (Tyr→Phe), 492 (Ile→Leu), 493 (Tyr→Gln), 494 (Arg→Ser), 498 (Tyr→Gln), 501 (Asp→Asn) and 505 (His→Tyr). The changes at positions 441, 486, 492, 493, 498 and 505 may not have any obvious effect on binding due to similar properties of amino acids.

### B-cell epitopes within RBD

Continuous B-cell epitopes were predicted using B-cell epitope prediction methods on IEDB server. The Kolaskar & Tongaonkar method predicted 11 antigenic epitopes in the RBD (Table 1), which can prompt B-cell responses. Surface accessibility analysis revealed four epitopes with surface accessibility (Table 1). Flexibility of epitopes is a measure of antigenicity [53]. The flexible epitopes in RBD were at positions 352–362, 380–392, 400–410, 421–433, 434–451, 454–473, 472–487 and 495–506. Beta-turns are the more flexible regions of the protein. According to Chou & Fasman predictions the beta-turn epitopes were at positions 437–443, 455–468, 422–428 and 495–500. Linear epitopes determined by BepiPred method are shown in Table 1. On the basis of consensus of all the methods, the peptides that can induce B-cell response were placed at positions 423–428, 455–461 and 494–500. The 423–428, 455–461 and 494–500, were the peptides that may prompt B-cell responses as predicted by ElliPro method. The mapping of epitopes on 3D structure of protein is shown in Supplementary Figure 2.

**Table 1.** Prediction of B-cell epitopes by Kolaskar & Tongaonkar, Emini surface accessibility and BepiPred methods.

S. No.	Start position	End position	Peptide	Peptide length
**Kolaskar & Tongaonkar method**				
1.	334	341	NLCPFGEV	8
2.	347	353	FASVYAW	7
3.	358	372	ISNCVADYSVLYNSA	15
4.	374	385	FSTFKCYGVSPT	12
5.	387	404	LNDLCFTNVYADSFVIRG	18
6.	407	412	VRQIAP	6
7.	429	436	FTGCVIAW	8
8.	455	461	LFRKSNL	6
9.	423	428	YKLPDD	5
10.	470	478	TEIYQAGST	9
11.	494	500	SYGFQPT	6
**Emini method**				
1.	419	428	ADYNYKLPDD	10
2.	437	442	NSNNLD	6
3.	455	468	LFRKSNLKPFERDI	14
4.	495	500	YGFQPT	6
**BepiPred method**				
1.	382	385	VSPT	4
2.	407	420	VRQIAPGQTGKIAD	14
3.	423	428	YKLPDD	6
4.	439	447	NNLDSKVGG	9
5.	461	463	LKP	3
6.	466	467	RD	2
7.	469	469	S	1
8.	473	483	YQAGSTPCNGV	11
9.	495	506	YGFQPTNGVGYQ	12

### Cytotoxic T-cell epitope prediction

The default setting in the NetCTL server was used to predict T-cell epitopes. On the basis of highest combinatorial scores, five epitopes (with NetCTL score = 1.1–2.5 nM) were opted for subsequent analysis (Table 2). On the basis of NetCTL scores the peptide with the highest score (2.5 nM) had sequence CVADYSVLY. Further analysis of all the five peptides for their binding with MHC-I showed that peptide CVADYSVLY illustrated binding with maximum MHC-I alleles (HLA-A*26:01, HLA-A*01:01, HLA-A*30:02, HLA-B*35:01, HLA-A*11:01, HLA-B*15:01, HLA-A*68:01, HLA-A*03:01, HLA-B*53:01 and HLA-C*07:01). The next peptide showing the binding with maximum number of alleles was FTNVYADSF.

**Table 2.** T-cell epitopes predicted to be recognized by MHC class I and class II alleles.

T-cell epitopes bind to specific MHC-I alleles		
S. No.	Peptide	MHC-I binding
1.	NATRFASVY	HLA-B*35:01 HLA-A*01:01
2.	RISNCVADY	HLA-A*30:02, HLA-B*15:01, HLA-A*03:01 and HLA-A*01:01
3.	CVADYSVLY	HLA-A*26:01, HLA-A*01:01, HLA-A*30:02, HLA-B*35:01, HLA-A*11:01, HLA-B*15:01, HLA-A*68:01, HLA-A*03:01, HLA-B*53:01 and HLA-C*07:01
4.	FTNVYADSF	HLA-A*01:01, HLA-B*15:01, HLA-B*15:03, HLA-B*15:17, HLA-A*25:01, HLA-A*26:01, HLA-B*08:03, HLA-B*58:01, HLA-B*53:01 and HLA-C*03:03
5.	ERDISTEIY	HLA-A*01:01

**T-cell epitopes bind to specific MHC-II alleles**		
**S. No.**	**Peptide**	**MHC-II binding**
1.	FELLHAPAT	*DRB1_0101 and DRB1_1001*
2.	FNATRFAS	*DRB1_0402*
3.	TGCVIAWNS	*DRB1_0403 and DRB3_0202*
4.	FRKSNLKPF	*DRB1_0701*
5.	YRLFRKSNL	*DRB1_0103, DRB1_0701, DRB1_0801, DRB1_0802, DRB1_1602, DRB4_0103, DRB1_1001, DRB1_1101, DRB1_1501, DRB4_0103 and DRB5_0101*
6.	VYAWNRKRI	*DRB1_1101, DRB1_1301, DRB4_0103, DRB5_0101, DRB1_0402 and DRB3_0202*
7.	FERDISTEI	*DRB3_0101*
8.	IRGDEVRQI	*DRB3_0101*
9.	VLYNSASFS	*DRB3_0202*

MHC-I: MHC class I; MHC-II: MHC class II.

### Helper T-cell epitope prediction

A total of nine peptides were predicted which exhibited strong affinity for MHC-II alleles (Table 2). Among these the peptide YRLFRKSNL and VYAWNRKRI reflected affinity for maximum number of alleles. YRLFRKSNL held strong affinity with large number of MHC-II allele including: *DRB1_0103, DRB1_0701, DRB1_0801, DRB1_0802, DRB1_1602, DRB4_0103, DRB1_1001, DRB1_1101, DRB1_1501, DRB4_0103 and DRB5_0101.*

### B- & T-cell epitopes feature profiling

To identify the best epitope for vaccine construction, different features of T-cell epitopes were determined (Table 3). The identified epitopes did not exhibit any homology with human proteins, were conserved and predicted to be nontoxic. The peptides which were digested by fewer enzymes have been considered good potential vaccine candidates (Table 3). Antigenicity of the peptides depicted that CTL specific peptides can be antigenic except the peptide ERDISTEIY. In case of helper T-cell epitopes, FELLHAPAT, TGCVIAWNS and VLYNSASFS were highly antigenic. In case of B-cell epitopes, all the three peptides were antigenic.

**Table 3.** Profiling of MHC class I, class II and B-cell specific peptides.

S. No.	Peptide	Homology with human proteins (Y/N)	Mutation (Y/N)	Toxicity (Y/N)	Hydrophobicity	Charge	Molecular weight	Antigenicity	Nondigesting enzymes
**MHC-I peptide**									
1.	NATRFASVY	N	N	N	-0.13	1.0	1028.24	1.03	Cyanogen bromide, idosobenzoate, proline endopept, staph protease, trypsin K and AspN
2.	RISNCVADY	N	N	N	-0.20	0.00	1040.27	1.07	Cymotrypsin, cyanogen bromide, trypsin K, endopept, staph protease andidosobenzoate
3.	CVADYSVLY	N	N	N	0.11	-1.00	1032.29	1.18	Trypsin, clostripain, cyanogen bromide, idosobenzoate, proline endopept, staph protease, trypsin K and trypsin R
4.	FTNVYADSF	N	N	N	0.03	-1.0	10632.24	1.03	Trypsin, clostripain, cyanogen bromide, idosobenzoate, proline endopept, staph protease, trypsin K and trypsin R
5.	ERDISTEIY	N	N	N	-0.30	-2.0	1125.30	0.95	Cymotrypsin, cyanogen bromide, idosobenzoate, proline endopept and trypsin K
**MHC-II peptide**									
1.	FELLHAPAT	N	N	N	0.10	-0.5	998.27	1.09	Trypsin, clostripain, cyanogen bromide, idosobenzoate, trypsin K, trypsin R and AspN
2.	FNATRFAS	N	N	N	-0.14	1.0	913.09	0.98	Cyanogen bromide, idosobenzoate, proline endopept, staph protease, N trypsin K and AspN
3.	TGCVIAWNS	N	N	N	0.11	0.0	950.20	1.05	Trypsin, clostripain, cyanogen bromide, proline endopept, staph protease, trypsin K, trypsin R and AspN
4.	FRKSNLKPF	N	N	N	-0.35	3.0	1136.48	0.99	Cyanogen bromide, idosobenzoate, staph protease and AspN
5.	YRLFRKSNL	N	N	N	-0.43	3.0	1196.54	0.99	Cyanogen bromide, idosobenzoate, proline endopept, staph protease and AspN
6.	VYAWNRKRI	N	N	N	-0.03	3.0	1205.55	0.99	Cyanogen bromide, proline endopept, staph protease and AspN
7.	FERDISTEI	N	N	N	-0.23	-2.0	1109.32	0.95	Cyanogen bromide, idosobenzoate, proline endopept and trypsin K
8.	IRGDEVRQI	N	N	N	-0.38	0.00	1085.36	0.98	Cymotrypsin, cyanogen bromide, idosobenzoate, proline endopept and trypsin K
9.	VLYNSASFS	N	N	N	0.06	0.00	987.19	1.09	Trypsin, clostripain, cyanogen bromide, idosobenzoate, proline endopept, staph protease, trypsin K, trypsin R and AspN
**B-cell epitopes**									
1.	YKLPDD	N	N	N	-0.34	-1.00	749.89	1.05	Clostripain, cyanogen bromide, idosobenzoate, staph protease, trypsin R and elastase
2.	LFRKSN	N	N	N	-0.44	2.00	763.97	1.03	Cyanogen bromide, idosobenzoate, proline endopept, staph protease and AspN
3.	SYGFQPT	N	N	N	-0.06	0.00	798.94	1.03	Trypsin, clostripain, cyanogen bromide, idosobenzoate, staph protease, trypsin K, trypsin R, elastase and AspN

Prediction of their toxicity, mutation, homologs present in human proteome, antigenicity, digestion with enzymes and other physiochemical properties as charge, weight and hydrophobicity.

MHC-I: MHC class I; MHC-II: MHC class II; N: No; Y: Yes.

### Interaction analysis of CTL epitopes with MHC-I specific alleles

**Figure 2.**
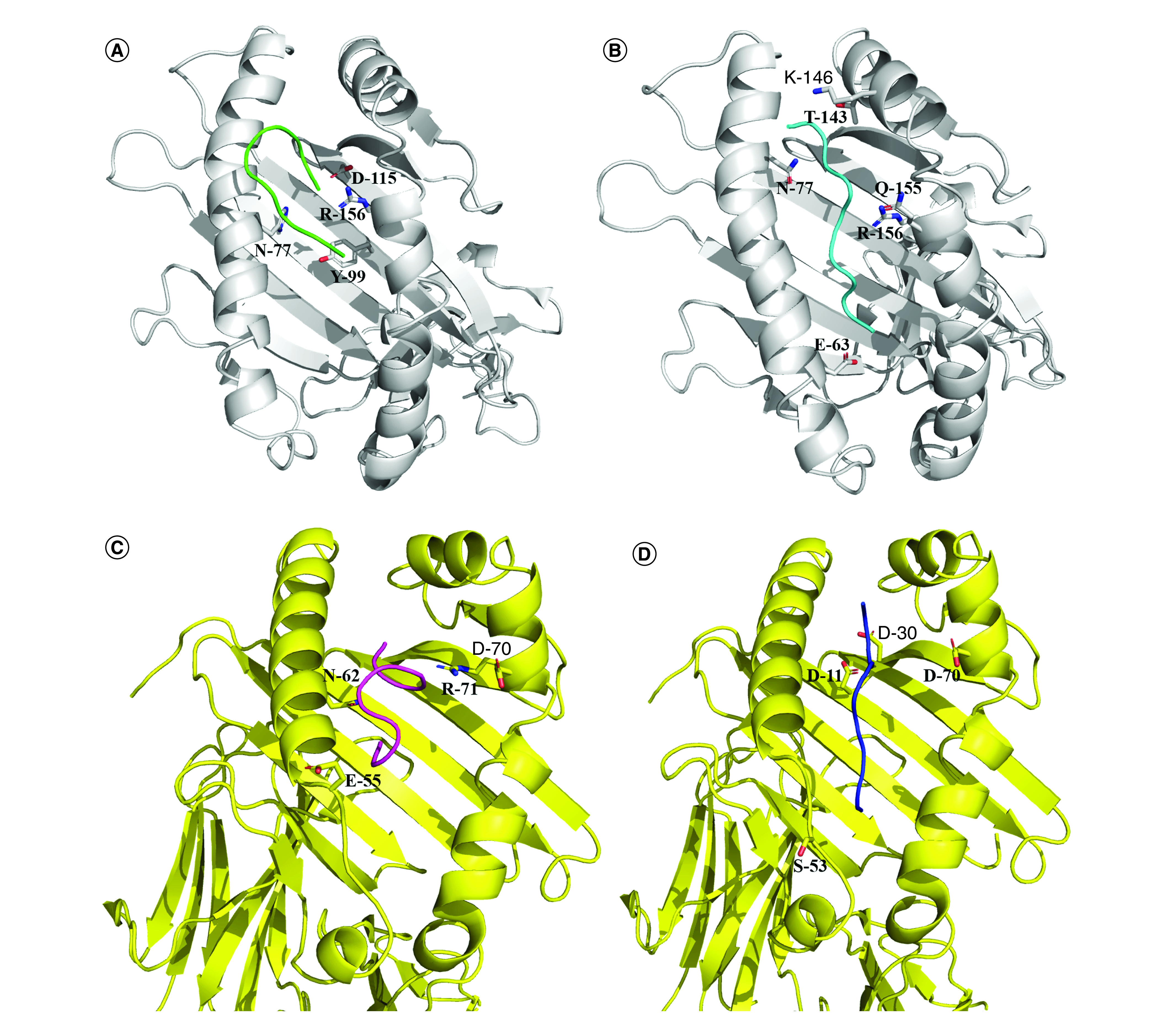
Interaction analysis of peptides with HLA-A & HLA-DR. **(A)** CVADYSVLY:HLA-A*01:01. **(B)** FTNVYADSF:HLA-A*01:01. **(C)** YRLFRKSNL:*DRB5_0101*. **(D)** VYAWNRKRI:*DRB5_0101*. HLA-A protein is shown in grey while HLA-DR protein is shown in yellow. The residues involved in interactions are highlighted as stick representation.

Two peptides (CVADYSVLY and FTNVYADSF) with the maximum number of bindings with HLA alleles were selected for interaction analysis. PEPFOLD created five models for each peptide and the model with the best score was selected for further analysis. HLA-A*0101 was selected as representative allele for interaction analysis. The docking of HLA-A with the top ranking peptide resulted in generation of 50 complexes for each peptide. The peptide 3 (CVADYSVLY) binds with HLA with binding energy of -11.3 KJ/mol, while a detailed interaction analysis revealed that peptide had H-bonding interactions with Asp-115, Arg-156, Tyr-99 and Asn-77 (Figure 2A). Peptide 4 (FTNVYADSF) bound with the HLA-A with an energy value of -12.6 KJ/mol. The peptide exhibited H-bonding interactions with Glu-63, Arg-156, Gln-155, Asn-77, Thr-143 and Lys-146 (Figure 2B).

### Interaction analysis of helper T lymphocyte epitopes with MHC-II specific alleles

Two of the peptides (YRLFRKSNL and VYAWNRKRI) that showed affinity with maximum number of MHC-II alleles were selected for interaction analysis. Docking of peptides with HLA-DRB5 depicted both the peptides to be binding with strong affinity. The peptide 6 and peptide 5 with maximum number of alleles were used to study affinity of these peptides with *DRB5_0101*. Peptide 5 (YRLFRKSNL) bound with the energy of -10.2 KJ/mol while peptide 6 (VYAWNRKRI) bound with the energy of -11.3 KJ/mol. The residues Asp-70, Asn-62, Arg-71 and Glu-55 of HLA-DRB5 were having H-bonding interactions with peptide 5 (Figure 2C). Interaction analysis revealed that Ser-53, Asp-11, Asp-30 and Asp-70 of HLA-DRB5 exhibited H-bonding interactions with peptide 6 (Figure 2D).

### Interaction analysis of BatCoV RaTG13 with bACE2

**Figure 3.**
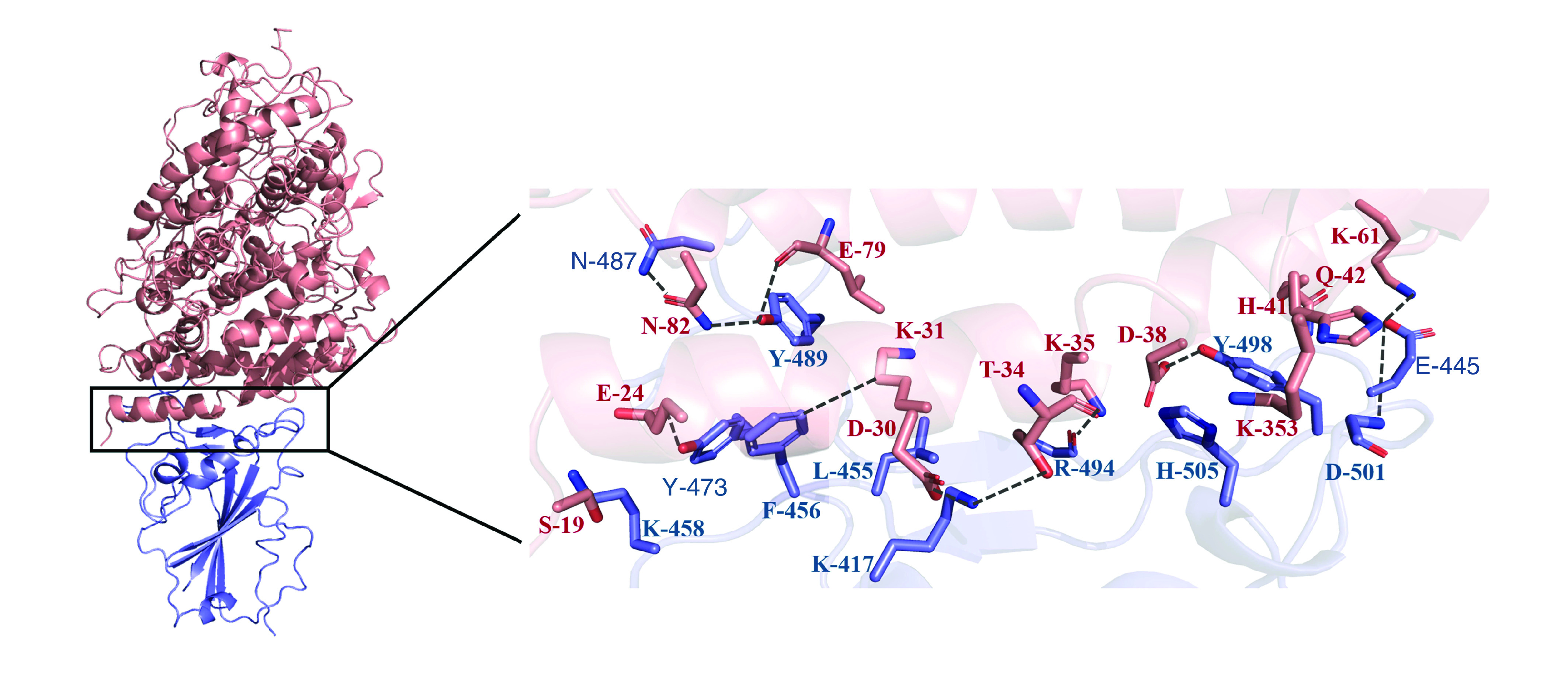
Top scoring complex of bat coronavirus RaTG13:bat ACE2. BatCoV RaTG13 is shown in blue while bACE2 is shown in pink. bACE2: Bat ACE2; BatCoV: Bat coronavirus.

The interactions of BatCoV RaTG13 with bACE2 were done using HADDOCK. There were 155 different complexes of BatCoV RaTG13:bACE2 that were generated, which clustered into 12 groups. Supplementary Table 1 shows the Z scores of all the seven clusters, size of each cluster, root-mean-square deviation (RMSD) from the overall lowest energy structure, and energy values of electrostatic, Van der Waals, and desolvation. The cluster with the best HADDOCK score (-178.9 ± 3.6) was further used for analysis. Detailed interaction analysis showed that 26 residues of bACE2 and nine residues of BatCoV RaTG13 were present at the interface. These residues were involved in 12 H-bonded, two salt bridges and 157 nonbonded contacts. The detailed interactions are shown in Table 4 and Figure 3.

**Table 4.** Interactions of bat coronavirus RaTG13 with bat angiotensin-converting enzyme2.

bACE2			BatCoV RaTG13	
S. No.	Residue name	Type of interactions	Residue name	Distance (Å)
1.	Glu-24	H-bond	Tyr-473	2.73
2.	Asp-30	H-bond	Lys-417	2.63
3.	Thr-30 Lys-353	H-bond	His-505	2.78
4.	Lys-35	H-bond	Arg-494	3.08
5.	Asp-38	H-bond	Tyr-498	2.55
6.	Gln-42	H-bond	Glu-445	2.73
7.	Lys-61	H-bond	Glu-445	2.86
8.	Leu-79	H-bond	Tyr-489	2.84
9.	Asn-82	H-bond	Asn-487	3.03
10.	Lys-31	H-bond	Leu-455 Phe-456	2.01 3.8
11.	Gln-42	H-bond	Tyr-489	3.4
12.	His-41	H-bond	Asp-501	3.9
13.	Asp-30	Salt bridge	Lys-417	2.63
14.	Lys-61	Salt bridge	Glu-445	2.77

bACE2: Bat ACE2; BatCoV: Bat coronavirus.

**Figure 4.**
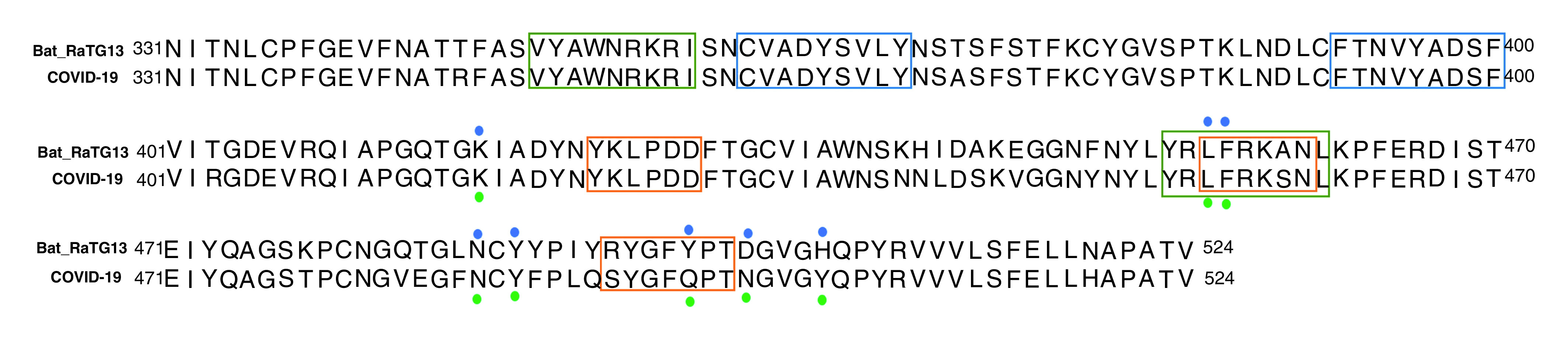
Sequence alignment of receptor binding domain of SARS coronavirus 2 with bat coronavirus RaTG13. B-cell epitopes are highlighted with orange boxes. CTL epitopes are highlighted with blue boxes. T-helper cell epitopes are highlighted as green boxes. Green dots represent SARS-CoV-2 interacting residues with hACE2 while blue dots represent interacting residues of BatCoV RaTG13 with bACE2. bACE2: Bat ACE2; BatCoV: Bat CoV; CoV: Coronavirus; CTL: Cytotoxic T lymphocyte; hACE2: Human ACE2.

The important residues of BatCoV RaTG13 that were involved in interactions with bACE2 are Lys-417, Leu-455, Phe-456, Asn-487, Tyr-489, Asp-501 and His-505. The important conserved residues of BatCoV RaTG13 that involved in interactions and demonstrated overlapping with epitopes were Leu-455 and Phe-456. These residues lay within the B-cell epitope (455-LFRKSN-461) and T-cell epitope (453-YRLFRKSNL-461). The important interacting residues of BatCoV RaTG13 with bACE2 and a comparison with SARS CoV2:hACE2 are shown in Figure 4.

## Discussion

Research on various CoV species earlier has been continually reported since the last one and a half decade after the emergence of SARS, for annotating signatures and virulence factors [80]. Viral entry receptors are crucial in viral life cycle, sustenance and egress [81]. Realizing their particular tissue tropism further augments their importance for therapeutic targeting and restricting viral entry into cell which can abolish infectivity altogether.

In case of SARS-CoV and MERS-CoV, S protein and specially S1 regions have been the prime focus in developing immune strategies against these strains [52]. Similar strategy can be employed by investigating the S protein for identifying immune epitopes against SARS-COV-2. Vaccines against SARS-CoV-2 can serve as one of the most promising modes of containing COVID-19 pandemic. To date no reliable treatment options are available for COVID-19, so logically vaccine against it is a much needed solution. As the global burden of infectivity by COVID-19 pandemic is increasing every day, computational biology aided vaccine design for SARS-CoV-2 with removal of unnecessary antigenic load and screened allergic response can provide the characteristic immune response required for preventing SARS-CoV-2 infection. Similar to SARS-CoV and MERS-CoV, S1 region of CVOID-19 harbors the RBD which is involved in the entry of virus into a host cell. Hence, the identification of antigenic peptides within RBD can be a good strategy to forestall infection. The present study focused on deriving immunogenic epitopes capable of triggering both humoral and cell mediated immune response, on the basis of high degree of comparative sequence similarity of RBD from BatCoV RaTG13 with SARS-CoV-2. Previously, immunogenic epitopes for SARS-CoV-2 by immunoinformatics method have been reported in comparison with SARS-CoV [23–25]. Using this approach may yield rather specific epitopes against SARS-CoV-2. Contextually, cross-reactivity of SARS-CoV antibodies against SARS-CoV-2 epitopes is also under debate and is providing useful information against potential SARS-CoV-2 host immune response [17,31,32].

Current results from analysis of RBD of SARS-CoV-2 revealed that 423-YKLPDD-428, 455-LFRKSN-461 and 494-SYGFQPT-500 are the B-cell epitopes that are highly antigenic. The two epitopes lie in receptor binding motif and also muddled in interactions with hACE2 [72]. The Leu-455, Phe-456, Ser-494 and Thr-500 were the conserved residues among all the SARS-CoV-2 sequences hooked in interactions with hACE2. In case of T-cell epitopes, several epitopes were identified from IEDB and NetCTL and the peptides manifesting affinity with maximum number of MHC-I/MHC-II alleles were further selected for analysis. Previously, it has been reported that peptides showing affinity with maximum number of HLA alleles could be very antigenic [53,54]. In case of CTL epitopes, two epitopes (peptide 3: CVADYSVLY and peptide 4: FTNVYADSF) were found to be highly antigenic and also showed strong binding with HLA-A allele. HLA are polymorphic proteins with variable expression in different population. Therefore, a vaccine which is suited for all population without inciting any autoimmunity met by for T-cell epitopes by HLA selectivity is crucial for an effective vaccine candidate [82]. In case of HLA-A*0101, the already docked peptide in crystal structure (PDB ID: 6AT9) has shown interactions with Tyr-7, Phe-9, Phe-33, Val-34, Tyr-59, Glu-63, Asn-77, Tyr-84, Ile-97, Tyr-99, Thr-143, Lys-146, Trp-147, Gln-155, Arg-156 and Tyr-159 of HLA-A [83]. Peptide 3 (CVADYSVLY) has paraded interactions with Arg-156, Tyr-99 and Asn-77 while peptide 4 (FTNVYADSF) showed interactions with maximum number of amino acids (Glu-63, Arg-156, Gln-155, Asn-77, Thr-143 and Lys-146). In case of helper T-cell epitopes, YRLFRKSNL and VYAWNRKRI illustrated affinity with maximum number of MHC-II alleles. One of the MHC-II allele HLA-DRB5*0101 was used to study possible interactions of peptide with HLA-DR. In case of HLA-DRB5*0101 (PDB ID: 1FV1) the crystallized structure displayed interactions with Asp-9, Phe-12, Glu-55, Met-23, Ser-53, Asn-62, Asp-66, Asn-69, Asp-11, Tyr-13, Asp-70, Arg-71, Tyr-78 and Asn-82 [84]. When we studied peptide 5 (YRLFRKSNL), Asn-62, Arg-71 and Glu-55 were residues important for binding of peptide. In case of peptide 6 (VYAWNRKRI), the Ser-53, Asp-11 and Asp-70 were involved in interactions with peptide. The interaction as well as binding energy data depicted these two peptides to be binding with HLA-DRB5 and can be used in vaccine construction. The peptide 453-YRLFRKSNL-461, present within the receptor binding motif and Leu-455 and Phe-456 illustrated interactions with hACE2 [72] as well as bACE2.

On the basis of different properties of these selected B- and T-cell epitopes, it can be observed that these epitopes were 100% conserved from the reported data, as predicted by conservation analysis. These peptides also did not exhibited homology with any human protein hence may not incite any autoimmunity. These peptides did not display any toxicity. The digesting enzyme data showed these peptides to be indigestible by a range of enzymes and hence are safer to use for vaccine development (Table 3). The B- and T-cell epitopes (LFRKSN and YRLFRKSNL) are overlapping, hence the presence of YRLFRKSNL in vaccine construct along with other peptides may enhance the efficacy of a vaccine. The analysis on the sequences submitted until October 2020 has shown that all the predicted epitopes have been fairly conserved except one change observed in T-cell epitope (453-YRLFRKSNL-461) region at position 453 as Y453F. This mutation was labeled as mink mutation and found in SARS-CoV-2 sequences obtained from Denmark, South Africa and The Netherlands [85,86]. The results proposed herein are preliminary and further *in vitro* and *in vivo* testing is required for the proposed vaccinable targets of SARS-CoV-2. Based on the study results, the predicted epitopes harbor attractive capability to be considered for ascertaining therapeutic potency. The identified epitopes from this study can further be investigated along with conserved region epitopes of S protein for molecular simulation studies, in construction of experimental vaccine constructs and for considering its potential as a peptide based vaccine.

Cumulatively, the detailed interaction analysis of BatCoV RaTG13 has shown that hydrophobic and charged residues have been involved in binding with bACE2. Analogous pattern has been observed in binding of SARS-CoV-2 with hACE2 receptor [72]. The important residues of BatCoV RaTG13 that were involved in interactions with bACE2 are Lys-417, Leu-455, Phe-456, Asn-487, Tyr-489, Asp-501 and His-505. The residues at these positions in SARS-CoV-2 were also involved in interactions with hACE2 [72]. Among them Lys-417, Leu-455, Phe-456, Asn-487 and Tyr-489 were the conserved residues (Figure 4).

Designing an effective vaccine against viral infection such as COVID-19 is tricky. On one hand, it has to be ensured that the vaccinable epitopes hold enough antigenic potential to mount a befitting yet specific immune response so as to rapidly clear the infection if the need arises; on the other hand, the host immune response should not be strong enough to trigger chronic inflammation which in case of COVID-19 might significantly deteriorate lung infection. Lung as an organ is highly sensitive to inflammatory changes initiated by a surge of cytokine response [87]. Mutations in the viral genome are capacitating CoVs to breach species barrier repeatedly. As the CoVs harbor an error-prone RNA dependent-RNA polymerase, it may engender recombination events with mutational diversity, concocting therapeutic challenges and survival advantage to the virus [80]. This has been the case observed in SARS-CoV epidemic of 2004 [88,89]. It is of grave concern that SARS-CoV-2 has the potential to reach pandemic proportions while considering the low persistent R_o_ estimates. As bats are considered primary hosts for CoV species, it will be interesting to scrutinize how bats evade viral entry as previous studies have identified bats evolving a mechanism for defying interferon pathway activation by the STING interferon pathway [90].

## Conclusion

The current study proposed the identification of potential multiple epitopes for vaccine development against SARS-CoV-2. The potential vaccine epitopes have been rigorously screened for multiple HLA, B-Cell, CTL and helper T lymphocyte epitopes thus augmenting its capability in inducing both humoral and cellular immune responses. Furthermore, it can be co-opted with adjuvant treatment in further enhancing viral disease control. The epitopes were further screened and validated for 100% conservancy with nonoverlapping human proteome thus additionally reducing the chances of autoimmunogenic side effects. The molecular docking of epitopes with HLA alleles were further validated for their mode of binding patterns and analysis of binding energy affinities.

**Summary points**

- The December of 2019 witnessed emergence of worldwide outbreak by a novel strain of coronavirus (CoV) termed SARS-CoV-2.
- There is no therapeutic or preventive strategy like vaccine developed so far to overcome infection.
- The receptor binding domain of SARS-Cov-2 for any potential vaccine epitopes were explored using immunoinformatics approach.
- The B-cell epitopes LFRKSN and SYGFQPT, were found to be highly antigenic. Among T-cell epitopes, the epitope CVADYSVLY and FTNVYADSF were antigenic and exhibited affinity for maximum number of MHC class I alleles. The T-cell epitopes YRLFRKSNL and VYAWNRKRI displayed affinity for maximum number of MHC class II alleles. The overlapping epitope among B- and T-cells was YRLFRKSNL.
- The epitopes were further screened and validated for 100% conservancy with nonoverlapping human proteome thus additionally reducing the chances of autoimmune side effects.
- The important conserved residues of BatCoV RaTG13, the Leu-455 and Phe-456 that have been involved in interactions, also demonstrated overlap with epitopes. These residues lay within the B-cell epitope (455-LFRKSN-461) and T-cell epitope (453-YRLFRKSNL-461).
- The deployment of these epitopes in potential vaccine against COVID-19 may help in sweeping the COVID-19 infectious spread.
