# Supplementary material for: Scouting the receptor-binding domain of SARS coronavirus 2: a comprehensive immunoinformatics inquisition
Source: Future Virol. 2021 Feb 22:10.2217/fvl-2020-0269. doi: 10.2217/fvl-2020-0269 (PMC7899787; doi:10.2217/fvl-2020-0269)
Supplement: Supplementary file 1 [file figure-s1.pdf]

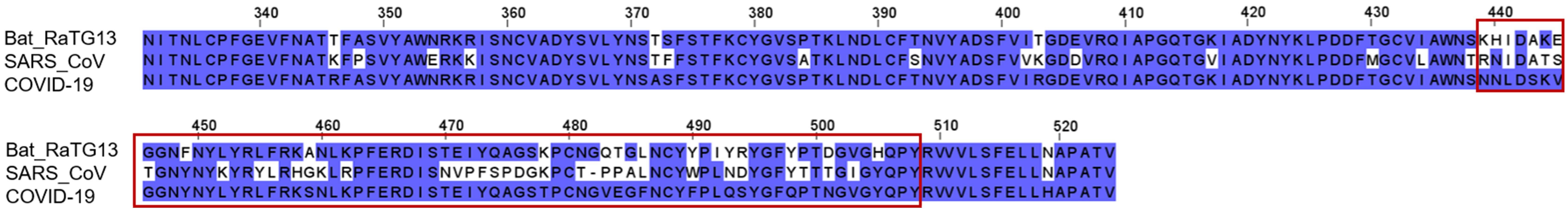
340 350 360 370 380 390 400 410 420 430 440
Bat_RaTG13 NITNLCPFGEVFNATTFASVYAWNRKRISNCVADYSVLYNSTSFSTFKCYGVSPTKLNDLCFTNVYADSFVITGDEVRQIAPGQTGKIADYNYKLPDDFTGCVIAWNSKHIDAKE
SARS_CoV NITNLCPFGEVFNATKFPSVYAWERKKISNCVADYSVLYNSTFFSTFKCYGVSATKLNDLCFSNVYADSFVVKGDDVRQIAPGQTGVIADYNYKLPDDFMGCVLAWNTRNIDATS
COVID-19 NITNLCPFGEVFNATRFASVYAWNRKRISNCVADYSVLYNSASFSTFKCYGVSPTKLNDLCFTNVYADSFVIRGDEVRQIAPGQTGKIADYNYKLPDDFTGCVIAWNSNNLDSKV
450 460 470 480 490 500 510 520
Bat_RaTG13 GGNFNYLYRLFRKANLKPFERDISTEIYQAGSKPCNGQTGLNCYYPIYRYGFYPTDGVGHQPYRVVVLSFELLNAPATV
SARS_CoV TGNYNYKYRYLRHGKLRPFERDISNVPFSPDGKPCT-PPALNCYWPLNDYGFYTTTGIGYQPYRVVVLSFELLNAPATV
COVID-19 GGNYNYLYRLFRKSNLKPFERDISTEIYQAGSTPCNGVEGFNCYFPLQSYGFQPTNGVGYQPYRVVVLSFELLHAPATV
