# Supplementary figures and images for: Scouting the receptor-binding domain of SARS coronavirus 2: a comprehensive immunoinformatics inquisition
Source: Future Virol. 2021 Feb 22:10.2217/fvl-2020-0269. doi: 10.2217/fvl-2020-0269 (PMC7899787; doi:10.2217/fvl-2020-0269)

**Epitope 3:**  
**494-500**  
**(SYGFQPT)**

**Epitope 2:**  
**455-461**  
**(LFRKSN)**

**Epitope 1:**  
**424-428 (KLPDD)**

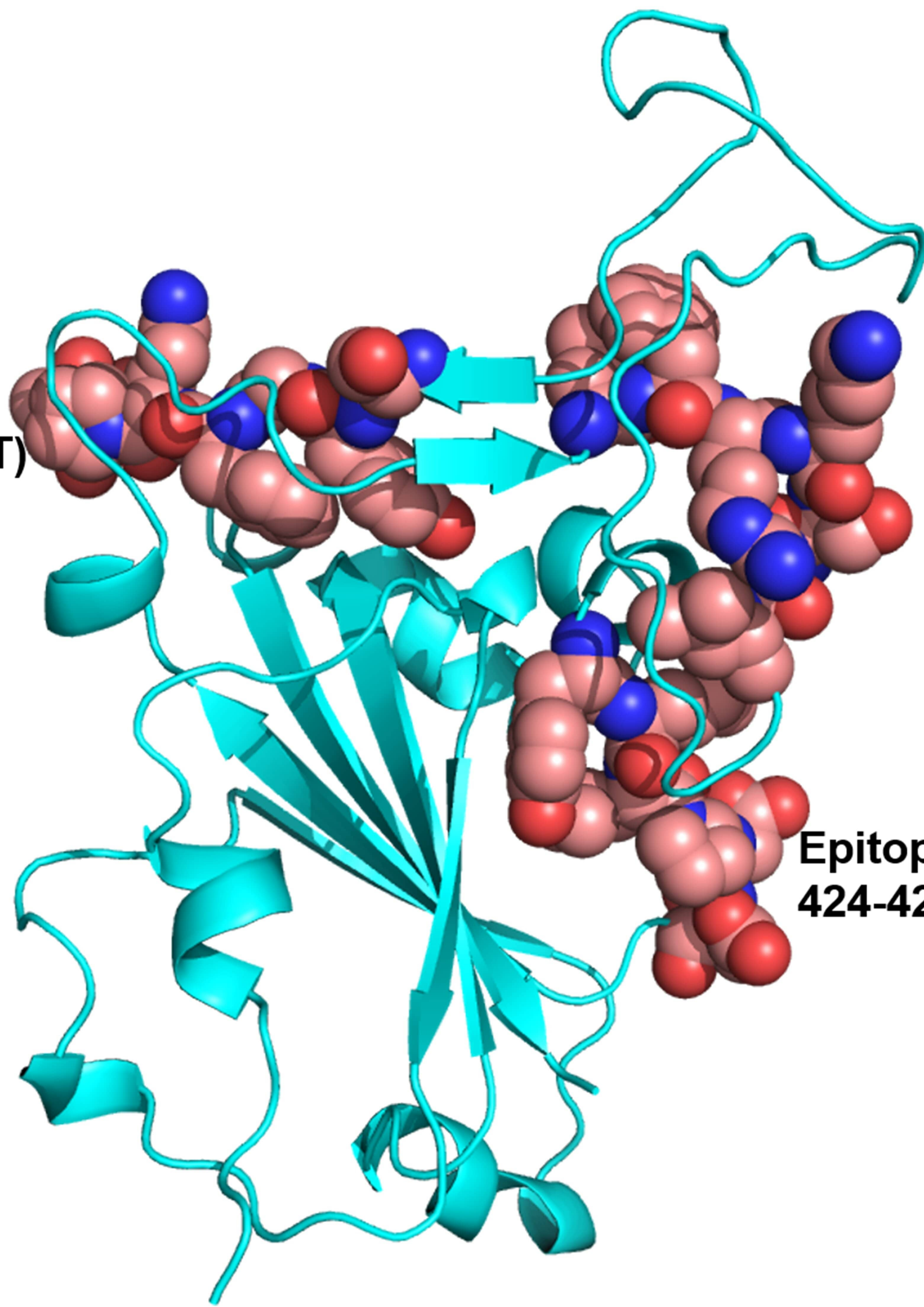

Supplement: Supplementary file 2 [file figure-s2.pdf]
