# Supplementary material for: Scouting the receptor-binding domain of SARS coronavirus 2: a comprehensive immunoinformatics inquisition
Source: Future Virol. 2021 Feb 22:10.2217/fvl-2020-0269. doi: 10.2217/fvl-2020-0269 (PMC7899787; doi:10.2217/fvl-2020-0269)
Supplement: Supplementary file 3 [file table-s1.docx]

| **Cluster No.** | **HADDOCK score** | **Cluster size** | **RMSD** | **Van der Waals energy (KJ/mol)** | **Electrostatic energy (KJ/mol)** | **Desolvation energy (KJ/mol)** | **Z-score** |
| --- | --- | --- | --- | --- | --- | --- | --- |
| 1 | -178.9 | 43 | 0.6 | -95.2 | -394.2 | -47 | -2.3 |
| 2 | -151.8 | 24 | 4.8 | -86.2 | -247.6 | -41.5 | -0.6 |
| 4 | -151.7 | 14 | 14.2 | -83.8 | -408.1 | -22.7 | -0.6 |
| 7 | -151.7 | 9 | 11.5 | -96.5 | -303.4 | -37.7 | -0.6 |
| 5 | -145.3 | 10 | 12.4 | -65.4 | -354.6 | -33.5 | -0.1 |
| 8 | -138.7 | 7 | 7.2 | -81 |  | -26.5 | 0.3 |
| 9 | -131.8 | 6 | 3.2 | -80.1 | -298.1 | -33.4 | 0.7 |
| 11 | -128.3 | 5 | 12.3 | -71.9 | -365.2 | -19.7 | 0.9 |
| 3 | -125.5 | 19 | 4.5 | -67.5 | -308.6 | -41.1 | 1.1 |
| 12 | -125.9 | 4 | 13.2 | -78.1 | -278.6 | -18.7 | 1.1 |

**Table S1:** Different energy values of bat-CoV-RaTG13:bACE2.
